# Identification of Pulmonary Hypertension Using Entropy Measure Analysis of Heart Sound Signal

**DOI:** 10.3390/e20050389

**Published:** 2018-05-21

**Authors:** Hong Tang, Yuanlin Jiang, Ting Li, Xinpei Wang

**Affiliations:** 1 Department of Biomedical Engineering, Dalian University of Technology, Dalian 116024, China; 2 College of Information and Communication Engineering, Dalian Minzu University, Dalian 116024, China; 3 School of Control Science and Engineering, Shandong University, Jinan 250100, China

**Keywords:** pulmonary hypertension, heart sound, sample entropy, fuzzy entropy, fuzzy measure entropy, kernel density estimation, confounding factor

## Abstract

This study introduced entropy measures to analyze the heart sound signals of people with and without pulmonary hypertension (PH). The lead II Electrocardiography (ECG) signal and heart sound signal were simultaneously collected from 104 subjects aged between 22 and 89. Fifty of them were PH patients and 54 were healthy. Eleven heart sound features were extracted and three entropy measures, namely sample entropy (SampEn), fuzzy entropy (FuzzyEn) and fuzzy measure entropy (FuzzyMEn) of the feature sequences were calculated. The Mann–Whitney U test was used to study the feature significance between the patient and health group. To reduce the age confounding factor, nine entropy measures were selected based on correlation analysis. Further, the probability density function (pdf) of a single selected entropy measure of both groups was constructed by kernel density estimation, as well as the joint pdf of any two and multiple selected entropy measures. Therefore, a patient or a healthy subject can be classified using his/her entropy measure probability based on Bayes’ decision rule. The results showed that the best identification performance by a single selected measure had sensitivity of 0.720 and specificity of 0.648. The identification performance was improved to 0.680, 0.796 by the joint pdf of two measures and 0.740, 0.870 by the joint pdf of multiple measures. This study showed that entropy measures could be a powerful tool for early screening of PH patients.

## 1. Introduction

Pulmonary hypertension (PH) is a hemodynamic and pathophysiological condition in which pulmonary artery pressure rises above a certain threshold. PH is a potentially fatal disease that can cause right heart failure [1]. If the PH is not diagnosed in a timely manner and no pretreatments are done actively, it will have serious consequences. In the early stage of PH, the symptoms are not apparent to be perceived by physicians but the mechanical activity of the heart has quietly changed and it can be reflected to some degree in the heart sound signals [2,3]. Therefore, the analysis of cardiac acoustic sound could play an important role in the initial diagnosis of PH.

Previous studies have shown that the time interval between the aortic component (A2) and the pulmonary component (P2) of the second heart sound (S2), as well as the dominant frequency of P2, increases in PH patients and they bring potential values to noninvasive diagnosis of PH [4,5]. The A2-P2 splitting interval (SI) was revealed to have links to pulmonary arterial pressure (PAP) [4,5,6]. Unfortunately, it was difficult to separate S2s, A2s, P2s from a heart sound recording reliably and precisely, as well as the splitting interval between A2 and P2 [7]. Some researchers turned their interests to extract features of the heart sounds that could provide relevant diagnostic information. For example, M Elgendi, P Bobhate and S Jain found useful features from time and frequency domains of a heart sound signal for distinguishing people with and without PH [8,9,10]. Another research team used machine learning algorithms to build models to classify people with and without PH based on features of the heart sounds [11]. Unlike the previous methods, this study introduced the entropy measures to analyze the heart sound signal of PH patients.

It is well-known that entropy is a quantitative measure of regularity of a sequence. It is reasonable to use entropy to investigate regularity differences of the heart sound features between people with and without PH. Eleven features of heart sounds were extracted and three entropy measures were used in this study to calculate their entropy values of each feature sequence. Typically, three entropy measures, i.e., sample entropy, fuzzy entropy and fuzzy measure entropy, were considered. Statistical analysis ranked the entropy measures according to the significance level. For identification purpose, the probability density function (pdf) of each entropy measure was built by kernel density estimation, as well as the joint pdf of two and multiple entropy measures. The results indicated that the entropy measures could be powerful to discriminate a PH patient from a healthy subject.

## 2. Materials and Methods

### 2.1. Data Collection

**Figure 1:**
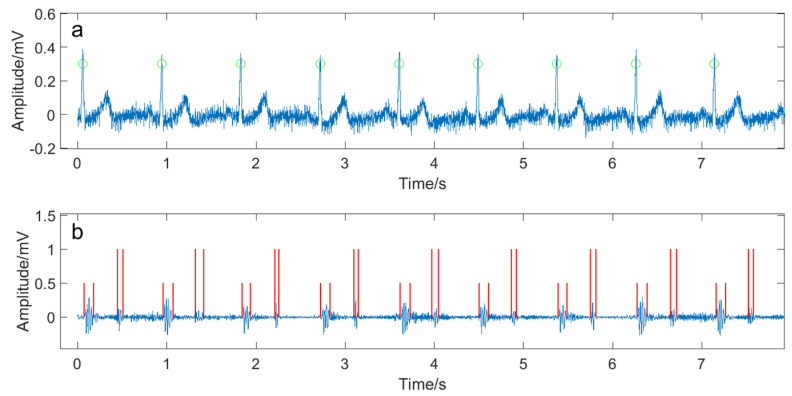
A part of collected signals from a PH subject. The subject was a female aged 76 with 158 cm in height and 64 kg in weight. Her pulmonary systolic pressure was 31 mmHg. (**a**) The ECG signal. The green circles showed the detected R waves; (**b**) the heart sound signal. The short and long red vertical lines indicated the detected S1s and S2s, respectively.

**Figure 2:**
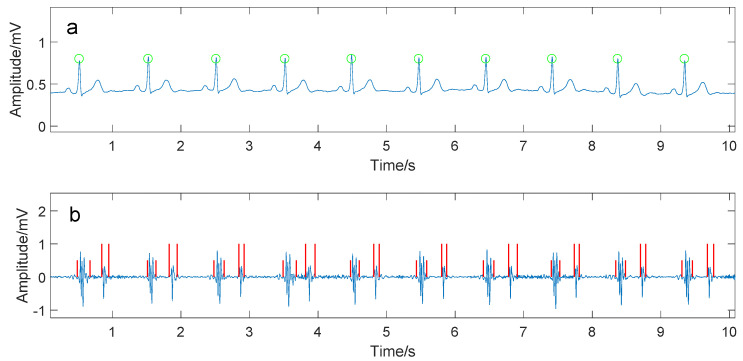
A part of collected signals from a healthy subject who was a male aged 67 with 165 cm in height and 63 kg in weight. (**a**) The ECG signal. The green circles showed the detected R waves; (**b**) the heart sound signal. The short red vertical lines indicated the detected S1s and the long ones showed the S2s.

One-hundred and four subjects participated in this study. All subjects were given informed and written consent. The involved subjects were divided into two groups. One was the PH group (50 patients in the second attached hospital of Dalian Medical University) and the other was the healthy control group (54 subjects enrolled from Dalian University of Technology and Shandong University). The basic information of the PH patient group and the healthy control group are shown in Table 1 and Table 2. A subject was asked to rest for 10 min prior to data collection. In the progress of data collection, each subject lied on his back quietly in a bed. The heart sound signal—Electrocardiography (ECG) lead II signal—were simultaneously recorded for at least five minutes at a sampling frequency of 2 kHz (PowerLab 8/35, ADInstruments, Sydney, Australia). A heart sound microphone sensor (MLT201, ADInstruments, Sydney, Australia) was placed at the left third intercostal space. The echocardiography was used to measure the pulmonary artery pressure for PH patients. The cardiologist in the hospital made the PH diagnose based on the measured data and symptoms. The PH patient group excludes those with atrial fibrillation or pacemaker. The subjects in the healthy control group were checked by ECG, echocardiography and a routine blood test to verify healthy condition. The R waves of the ECG signals, picked up by the Pan–Tomkins algorithm [12], were used to determine the beginning of a cardiac cycle. The first heart sound (S1) and the second heart sound (S2) were segmented by the Shannon energy envelope [13]. A manual interference was necessary to verify the segmentation if the envelope-based segmentation algorithm failed, especially for the PH patients’ heart sound signals where heavy murmurs often occurred. Figure 1 and Figure 2 were two examples of heart sound segmentation for a PH subject and a healthy subject.

### 2.2. Feature Extraction

**Figure 3:**
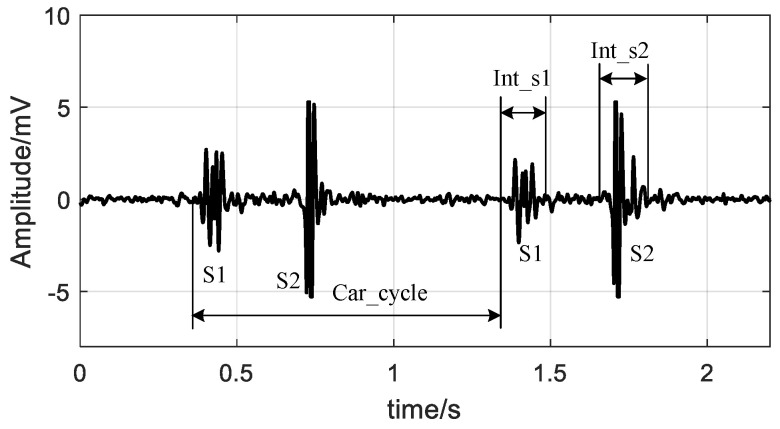
Illustration of the definition of “Int_s1”, “Int_s2”, and “Car_cycle”.

The time interval of a heart sound and cycle duration were considered as features in this study. The sound interval was the time duration from the start to the end of the heart sound, and the cycle duration was the time duration from the start of S1 to the start of the next S1, as illustrated in Figure 3. The authors defined “Int_s1” as the time interval of S1, “Int_s2” as the time interval of S2, and “Car_cycle” as the cardiac cycle duration. An “Int_s1”, an “Int_s2” and a “Car_cycle” can be detected from one cardiac cycle signal of the heart sound recording. So, feature sequences of “Int_s1”, “Int_s2” and “Car_cycle” were produced from a heart sound recording.

**Figure 4:**
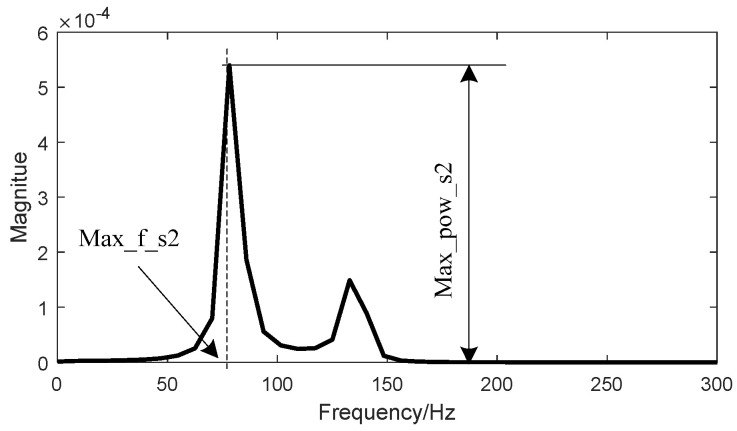
Illustration of features extracted from power spectral domain. “Max_pow_s2” was the maximum magnitude of power spectral density of the second heart sound and “Max_f_s2” was the corresponding frequency.

Another four features were from the power spectral domain of S1 and S2. The Burg algorithm was used to calculate the power spectral density (PSD) because of its smoothing character where the order of the autoregressive model was empirically set as 4 [14]. The maximum magnitude of the power spectral density of S2, denoted as “Max_pow_s2” and the corresponding frequency value, denoted as “Max_f_s2” were extracted as two features, as illustrated in Figure 4. The “Max_pow_s1”and “Max_f_s1” were extracted in a similar way.

Another four features considered in energy domain were the average energy of S1 (denoted as “Ener_s1”), the average energy of S2 (denoted as “Ener_s2”), the average Shannon energy of S1 (denoted as “ShanEner_s1”) and the average Shannon energy of S2 (denoted as “ShanEner_s2”), as given in the following

(1) $$\mathrm{Ener}\_S1=\frac{1}{L_{s1}}\sum_{i=1}^{L_{s1}}{\left(s_{1}\left(i\right)\right)}^{2}$$

(2) $$\mathrm{Ener}\_S2=\frac{1}{L_{s2}}\sum_{i=1}^{L_{s2}}{\left(s_{2}\left(i\right)\right)}^{2}$$

(3) $$\mathrm{ShanEner}\_S1=-\frac{1}{L_{s1}}\sum_{i=1}^{L_{s1}}{\left(s_{1}\left(i\right)\right)}^{2}*\log[{\left(s_{1}\left(i\right)\right)}^{2}]$$

(4) $$\mathrm{ShanEner}\_S2=-\frac{1}{L_{s2}}\sum_{i=1}^{L_{s2}}{\left(s_{2}\left(i\right)\right)}^{2}*\log[{\left(s_{2}\left(i\right)\right)}^{2}]$$

where $s_{1}(i)$ and $s_{2}(i)$ were the S1 and S2 detected by the envelope-based algorithm. $L_{s1}$ and $L_{s2}$ were the number of sampling points of the heart sounds.

**Figure 5:**
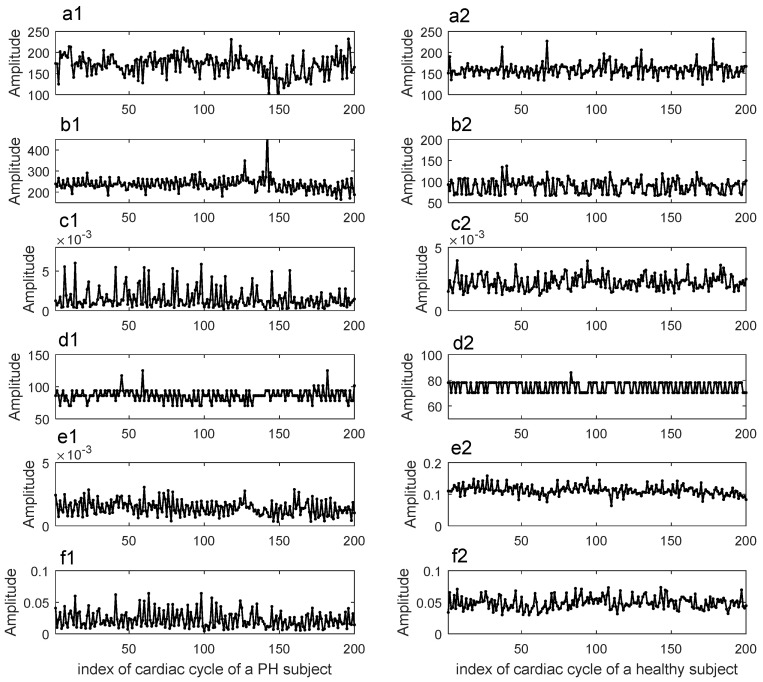
An example to illustrate some feature sequences of a PH patient and a healthy subject. The PH patient was a 76-year-old female of 158 cm in height and 64 kg in weight. Her pulmonary systolic blood pressure was 31 mmHg. The healthy subject was a 67-year-old male of 165 cm in height and 63 kg in weight. (**a1**,**a2**) Sequence of Int_s1 in ms; (**b1**,**b2**) sequence of Int_s2 in ms; (**c1**,**c2**) sequence of Max_pow_s2; (**d1**,**d2**) sequence of Max_f_s2 in Hz; (**e1**,**e2**) sequence of Ener_s1; and (**f1**,**f2**) sequence of Ener_s2.

The 11 features used in this study were summarized in Table 3. An example to illustrate some of the feature sequences extracted from a PH subject and a healthy subject were given in Figure 5.

### 2.3. Entropy

#### 2.3.1. Sample Entropy

As a measure of the complexity of a digital sequence, the entropy measures have been widely used in physiological signals such as electrocardiogram signals, heart rate variability signals and heart sound signals [15,16,17]. To quantify the regularity of the short and noisy physiological time series, approximate entropy (ApEn) was firstly proposed by Pincus [18]. The calculation of ApEn was very easy and it was quickly applied to the various clinical cardiovascular studies [19,20]. However, ApEn lacked consistency and the results depended on data length. In the meanwhile, it could cause a biased estimation for the complexity of physiological signals because of self-matching. In order to overcome the defects above, Richman and Moorman proposed the sample entropy (SampEn) [21], which was relatively consistent and dependent on data length less. Here SampEn was used to calculate the entropy value of a feature sequence as an entropy measure. The algorithm to calculate SampEn is as the follows.

For a feature sequence x(i) (1≤i≤N), given the embedding dimension m and threshold parameter r to form the vector $X_{i}^{m}$

(5) $$X_{i}^{m}=\left\{x(i),x(i+1),\cdots ,x(i+m-1)\right\},\;1\leq i\leq N-m,$$

where N is the number of feature samples. The vector $X_{i}^{m}$ represents m consecutive samples of x(i) started from index i. Let $d_{i,j}^{m}$ denote the distance between $X_{i}^{m}$ and $X_{j}^{m}$ based on the maximum absolute difference

(6) $$d_{i,j}^{m}=d\left(X_{i}^{m},X_{j}^{m}\right)=\max_{k=0}^{m-1}\left|x(i+k)-x(j+k)\right|,$$

where d(.) is the function to calculate the maximum absolute difference. Then $B_{i}^{m}(r)$ can be calculated as

(7) $$B_{i}^{m}(r)=\sum_{j=1,j\neq i}^{N-m}A(r-d_{i,j}^{m})/(N-m-1),$$

where A(⋅) is the Heaviside function

(8) $$A(r-d_{i,j}^{m})=\left\{ \begin{array}{l} 1,d_{i,j}^{m}\leq r\\ 0,d_{i,j}^{m}>r \end{array}\right.,$$

(9) $$B^{m}(r)=\ln\left[\sum_{i=1}^{N-m}B_{i}^{m}(r)/\left(N-m\right)\right].$$

Then increase m by 1 and repeat the steps above to get $B^{m+1}(r)$. Finally, SampEn is defined by

(10) $$SampEn(m,r,N)=B^{m}(r)-B^{m+1}(r).$$

In this study, the embedding dimension m = 2 and threshold parameter r is set to be 0.2 times of the sequence’ standard deviation [20].

#### 2.3.2. Fuzzy Entropy

SampEn is based on the Heaviside function of the classical sets which is a two-state classifier that judges two vectors as either ‘‘similar’’ or ‘‘dissimilar’’ with no intermediate states and which influences the statistical stability of results. To enhance the statistical stability, another entropy named fuzzy entropy (FuzzyEn) was proposed and the Heaviside function was replaced by the Zadeh fuzzy sets that provided a smooth similarity classifier [22,23]. The FuzzyEn and the SampEn algorithm are basically the same, except that (7) and (8) are replaced by

(11) $$B_{i}^{m}(r)=\sum_{j=1,j\neq i}^{N-m}A(d_{i,j}^{m})/(N-m-1),$$

(12) $$A(d_{i,j}^{m})=\exp(-\ln{\left(d_{i,j}^{m}/r\right)}^{2}).$$

Finally, FuzzyEn is defined by

(13) $$FuzzyEn(m,r,N)=B^{m}(r)-B^{m+1}(r).$$

In this study, the embedding dimension m = 2 and threshold parameter r is set to be 0.2 times of the sequence’ standard deviation [20].

#### 2.3.3. Fuzzy Measure Entropy

Fuzzy entropy focuses only on the local characteristics of the sequence and the global fluctuation may affect the results. In order to combine both the local and global similarity of the time series, the fuzzy measure entropy (FuzzyMEn) method was proposed [24], which is described as follows.

For a feature sequence x(i) (1≤i≤N), given the embedding dimension m to form the local vector $XL_{i}^{m}$ and the global vector $XG_{i}^{m}$

(14) $$\begin{array}{l} XL_{i}^{m}=\left\{x(i),x(i+1),\cdots ,x(i+m-1)\right\}-\bar{x}(i)\\ XG_{i}^{m}=\left\{x(i),x(i+1),\cdots ,x(i+m-1)\right\}-\bar{x} \end{array},\;1\leq i\leq N-m.$$

The vector $XL_{i}^{m}$ represents m consecutive samples in x(i) starting from the index i and it removes the local baseline $\bar{x}(i)$, which is defined as

(15) $$\bar{x}(i)=\frac{1}{m}\sum_{k=0}^{m-1}x(i+k),\;1\leq i\leq N-m.$$

The vector $XG_{i}^{m}$ indicates m consecutive samples in x(i) from the index i and it removes the global mean value $\bar{x}$ of x(i), which is defined as:

(16) $$\bar{x}=\frac{1}{N}\sum_{i=1}^{N}x(i).$$

Then the distance of the local sequence between $XL_{i}^{m}$ and $XL_{j}^{m}$ is defined as $dL_{i,j}^{m}$ and the distance of the global sequence between $XG_{i}^{m}$ and $XG_{j}^{m}$ is defined as $dG_{i,j}^{m}$. The $dL_{i,j}^{m}$ and $dG_{i,j}^{m}$ are calculated as

(17) $$\begin{array}{l} dL_{i,j}^{m}=d\left(XL_{i}^{m},XL_{j}^{m}\right)=\max_{k=0}^{m-1}\left|\left(x(i+k)-\bar{x}(i))-(x(j+k)-\bar{x}(j)\right)\right|\\ dG_{i,j}^{m}=d\left(XG_{i}^{m},XG_{j}^{m}\right)=\max_{k=0}^{m-1}\left|\left(x(i+k)-\bar{x})-(x(j+k)-\bar{x}\right)\right| \end{array}.$$

Then compute the local similarity degree $DL_{i,j}^{m}(n_{L},r_{L})$ between $XL_{i}^{m}$ and $XL_{j}^{m}$ by the fuzzy function $\mu L(dL_{i,j}^{m},n_{L},r_{L})$, and calculate the global similarity degree $DG_{i,j}^{m}(n_{G},r_{G})$ between $XG_{i}^{m}$ and $XG_{j}^{m}$ by the fuzzy function $\mu G(dG_{i,j}^{m},n_{G},r_{G})$.

(18) $$\begin{array}{l} DL_{i,j}^{m}(n_{L},r_{L})=\mu L(dL_{i,j}^{m},n_{L},r_{L})=\exp(-{\left(dL_{i,j}^{m}\right)}^{n_{L}}/r_{L})\\ DG_{i,j}^{m}(n_{G},r_{G})=\mu G(dG_{i,j}^{m},n_{G},r_{G})=\exp(-{\left(dG_{i,j}^{m}\right)}^{n_{G}}/r_{G}) \end{array},$$

where the $r_{L}$ and $r_{G}$ are the thresholds, which are both set as 0.2 times of the sequences’ standard deviation [20]. The $n_{L}$ and $n_{G}$ are weights of sequences’ similarity, which are set to be 3 and 2, respectively [24]. Define the function $\phi L^{m}(n_{L},r_{L})$ and $\phi G^{m}(n_{G},r_{G})$ as

(19) $$\begin{array}{l} \phi L^{m}(n_{L},r_{L})=\frac{1}{N-m}\sum_{i=1}^{N-m}(\frac{1}{N-m-1}\sum_{j=1,j\neq i}^{N-m}DL_{i,j}^{m}(n_{L},r_{L}))\\ \phi G^{m}(n_{G},r_{G})=\frac{1}{N-m}\sum_{i=1}^{N-m}(\frac{1}{N-m-1}\sum_{j=1,j\neq i}^{N-m}DG_{i,j}^{m}(n_{G},r_{G})) \end{array},\;1\leq i,j\leq N-m.$$

Increase m by 1 and repeat the steps above to get $\phi L^{m+1}(n_{L},r_{L})$ and $\phi G^{m+1}(n_{G},r_{G})$. Then the fuzzy local measure entropy (FuzzyLMEn) and the fuzzy global measure entropy (FuzzyGMEn) are defined as

(20) $$\begin{array}{l} FuzzyLMEn(m,n_{L},r_{L},N)=-\ln(\phi L^{m+1}(n_{L},r_{L})/\phi L^{m}(n_{L},r_{L}))\\ FuzzyGMEn(m,n_{G},r_{G},N)=-\ln(\phi G^{m+1}(n_{G},r_{G})/\phi G^{m}(n_{G},r_{G})) \end{array}.$$

Finally, FuzzyMEn is defined as

(21) $$\begin{array}{l} FuzzyMEn(m,n_{L},r_{L},n_{G},r_{G},N)=FuzzyLMEn(m,n_{L},r_{L},N)\\ \;+FuzzyGMEn(m,n_{G},r_{G},N) \end{array}.$$

This study used the three entropies to measure the regularity difference of a feature sequence between PH patients and healthy subjects. Eleven feature sequences were extracted from a heart sound recording and sample entropy, fuzzy entropy and fuzzy measure entropy were calculated for each feature sequence. In total, three entropies combined with 11 feature sequences yield 33 entropy measures for a heart sound recording. For example, the sample entropy measure of a Max_pow_s2 sequence is abbreviated as SampEn_Max_pow_s2. Similarly, the fuzzy entropy measure of a Max_pow_s2 sequence is abbreviated as FuzzyEn_Max_pow_s2.

### 2.4. Statistical Tests

It is known from the mechanism of heart sound generation that the proposed heart sound features reflect physiological and pathological condition of heart hemodynamics. So, the complexity embedded in a heart sound feature sequence, measured by the entropy, will vary by body condition. The entropy value is hopefully different in different body condition even for the same subject. On the other hand, the estimated entropy value also changes in noisy environments. The authors in this study took the entropy values as random numbers and made an assumption that the entropy values had different distribution for PH subject and healthy subject. A question arises as to how to evaluate the significance of an entropy measure between the PH group and the healthy group. In this study, the Mann–Whitney U test was used to achieve this purpose. The Mann–Whitney U test is a nonparametric test applicable to non-Gaussian distribution data. Based on this test, one can draw a conclusion that any two entropy measures come from the same group as long as they have an equal median [25]. The significance level of each entropy measure calculated by the test was used. In a typical case, a threshold of 0.05 is set for the significance level. However, this study considered multiple tests of statistical significance on the same data. As such, a Bonferroni correction was further used to improve the significance test. If the significance level was less than the threshold, it is unlikely that the two entropy measures are from the same group, i.e., they are from the PH group and health group, respectively.

### 2.5. Probability Density Function of an Entropy Measure Fitted by Kernel Density Estimation

To characterize the random numbers, it is necessary to build the probability density function (pdf). For a single entropy measure, its pdf can be constructed by the nonparametric kernel density estimation (KDE) based on a Gaussian kernel function [26]. Suppose e(i) (1≤i≤n) is an entropy measure of a feature sequence and n is the number of a subject group. The pdf of an entropy measure can be estimated by

(22) $$\hat{f}\left(e\right)=\frac{1}{nh}\sum_{i=1}^{n}K_{1}\left(\frac{e-e(i)}{h}\right),$$

where h is a smoothing parameter called bandwidth and $K_{1}(\cdot )$ is the single-variable kernel function

(23) $$K_{1}(\mu )=\frac{1}{\sqrt{2\pi }}\exp\left(-\frac{1}{2}\mu^{2}\right).$$

The Silverman’s rule of thumb for the bandwidth is used to get the best one [27]

(24) $$h=1.06\sigma n^{-1/5}.$$

A joint pdf of multiple entropy measure can be generalized from (20). Let $e=[e_{1}\;e_{2}\;\cdots \;e_{d}]$ be a d-dimensional entropy measure vector. The joint pdf $f^{d}(e)$ of the entropy measures can be obtained by

(25) $$f^{d}(e)=\frac{1}{n}{\left|H\right|}^{-1/2}\sum_{i=1}^{n}K_{2}\left({\left|H\right|}^{-1/2}(e-e_{i})\right),$$

where $e_{i}={\left(e_{i1},e_{i2},\ldots ,e_{id}\right)}^{T}$, i=1,2,…n. $K_{2}(\cdot )$ is the multiple-variable kernel function which is a symmetric multivariate density. The standard multivariate normal kernel was used here.

(26) $$K_{2}(\mu )={\left(2\pi \right)}^{-d/2}|H{|}^{-1/2}\exp\left(-\frac{1}{2}\mu^{T}H^{-1}\mu \right),$$

where μ is a *d*-dimensional vector. H is the d×d bandwidth (or smoothing) matrix which is symmetric and positive definite. Similarly, the Silverman’s rule of thumb gives the best bandwidth matrix H [27,28,29]:

(27) $$\sqrt{H_{ii}}={\left(\frac{4}{d+2}\right)}^{\frac{1}{d+4}}n^{\frac{-1}{d+4}}\sigma_{i},$$

where $\sigma_{i}$ is the standard deviation of the *i*-th variable and $H_{ij}=0,i\neq j$.

### 2.6. Identification of a PH Patient from a Healthy Subject Using the pdf Based on the Bayes’ Decision Rule

The pdf of PH group and the health control group were estimated by Section 2.5. If the Mann–Whitney U test shows that an entropy measure is significant between the two groups, the pdf of the entropy measure for PH group must be somewhat different from that of health group. The authors proposed algorithms to identify an unknown subject based on Bayes’ decision rule, as seen in Table 4 and Table 5.

An unknown subject could be classified correctly or incorrectly. So, four cases may occur in the results. A case that a PH subject is correctly identified as a PH patient is called true positive (TP). A case that a healthy subject wrongly is wrongly identified as a PH patient is called false positive (FP). A case that a healthy subject is correctly identified as a healthy subject is called true negative (TN). A case that a PH subject is wrongly identified as a healthy subject is called false negative (FN). Using the above identification algorithms, an unknown subject was identified. The number of TP, FN, TN and FP are defined as *num*_*TP, num*_*FN, num*_*TN,* and *num*_*FP*, respectively. The sensitivity and specificity is therefore calculated as [30]

(28) $$Sen=\frac{num\_TP}{num\_TP+num\_FN},$$

(29) $$Spe=\frac{num\_TN}{num\_TN+num\_FP}$$

where Sen and Spe represent the values of sensitivity and specificity. Then the overall evaluation index was defined by the accuracy to measure the identification performance

(30) $$Acc=\frac{num\_TP+num\_TN}{num\_TP+num\_TN+num\_FP+num+FN}.$$

## 3. Results and Discussions

### 3.1. Significance of the Features and Reduction of the Age Confounding Factor

To investigate the significance of the 33 entropy measures, the Mann–Whitney U test was performed, as shown in Table 6. The least *p* value, 1.20 × 10^−^^9^, was of SampEn_max_pow_s2 (No. 13). The measures related to max_pow_s2 had very low *p* values around 10^−9^ E-09 (No. 13–No. 15). The *p* values of the measures from No. 1 to No. 30 were small. The maximum *p* value, 4.22 × 10^−^^1^, was of SampEn_Int_s1 (No. 31). The *p* values of entropy measures related to Int_s1 (No. 31–33) were much greater than others, which meant that these measures were not significant between PH and healthy group.

In this study, the average age of PH group and health group was 69.4 and 32.6, respectively, as seen in Table 1 and Table 2. There was significant difference. There is doubt whether the significance of the proposed measures is likely attributed to age difference or not. As such, age is a confounding factor. To investigate the effect of the age confounding factor, the authors performed Pearson correlation analysis between the measures and the age values. The correlation coefficients (CC.) were given in Table 6. The measure, SampEn_max_f_s2 (No. 16), had maximum correlation coefficient of 0.43. The minimum correlation coefficient came from FuzzyEn_max_f_s1 (No. 8), 0.14. A further check showed that the measures from No. 12 to No. 29 had somewhat high correlation coefficients. The correlation analysis showed that the age did contribute measure difference between the two groups to some degree. To reduce the age confounding factor, it is reasonable to discard those measures which have high correlation coefficients with age. The authors set a threshold, 0.30, for the coefficients. The measures with absolute coefficients less than 0.30 are believed to be weakly correlated with age. Therefore, nine measures indicated by bold text (No. 1, 2, 4, 5, 6, 7, 8, 9 and 11) were selected as candidate measures for identification purpose.

A threshold for significance level is usually set as 0.05. However, it is known that a Bonferroni correction is a safeguard against multiple tests of statistical significance on the same data [31]. So, the significance level in this study was set as 0.05/9, i.e., 5.6 × 10^−^^3^. That is to say, an entropy measure will be safely significant if its significance level is less than 5.6 × 10^−^^3^. It was found that the selected nine measures (except No. 9 which was close to the threshold) were highly significant between the two groups. It revealed that the selected nine measures were unlikely from the same group. They were reasonable to be used for identification.

### 3.2. Identification Performance of a Single Entropy Measure

**Figure 6:**
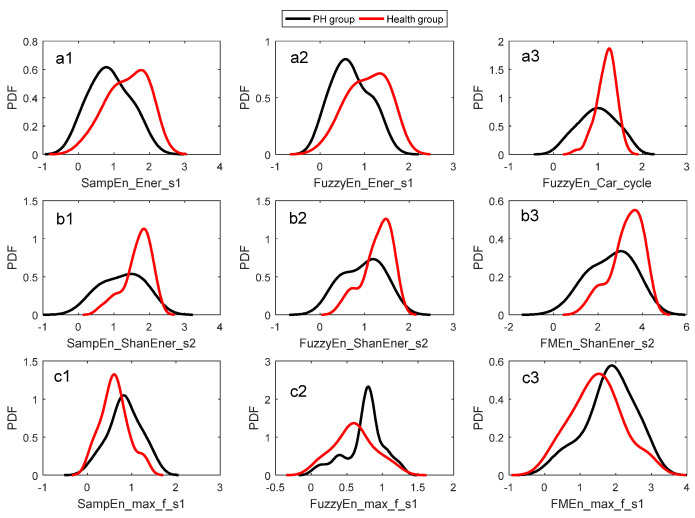
Estimated pdf pairs. (**a1**,**a2**) The two pdf pairs of Ener_s1; (**a3**) the pdf pairs of Fuzzy entropy of cardiac cycle; (**b1**–**b3**) the three pdf pairs of ShanEner_s2; (**c1**–**c3**) the three pdf pairs of Max_f_s1.

**Figure 7:**
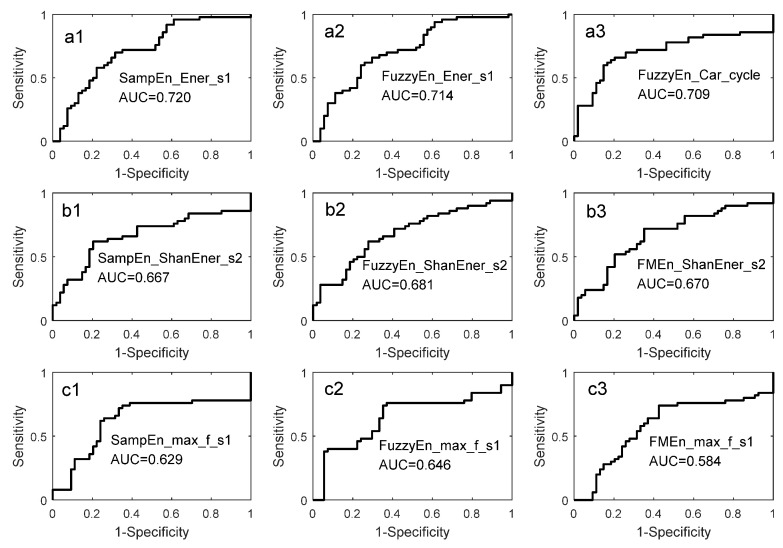
ROC curve of a single entropy measure. (**a1**,**a2**) the ROC curves of Ener_s1; (**a3**) the ROC curve of cardiac cycle; (**b1**–**b3**) the ROC curves of ShanEner_s2; (**c1**–**c3**) the ROC curves of Max_f_s1.

For a selected entropy measure, both pdfs of the PH group and the health control group were fitted respectively by KDE as given in Section 2.5. The leave-one-out cross-validation was used to evaluate the identification performance. That is, one subject was taken out and the other was involved in pdf estimation. The curves of estimated pdf pairs were shown in Figure 6. The red and black curves were for health group and PH group, respectively. A subject can be identified by the Algorithm 1 proposed in Section 2.6. The corresponding Receiver Operating Characteristic (ROC) curves were given in Figure 7. The summary of identification performance was showed in Table 7 in term of sensitivity, specificity, accuracy and area under curve (AUC).

It was seen in Table 7 that the entropy measures related to Ener_s1 had the best identification performance whose AUC was higher than 0.70. For example, the sensitivity, specificity and accuracy of SampEn_Ener_s1 was 0.720, 0.648 and 0.683. The performance of entropy measures related to Max_f_s1 was the worst. The identification performance of the entropy measures can also be highly reflected by the overlapping of the pdf pairs. A careful check to the pdf pairs shown in Figure 6 may indicate that the pdf pairs related to Ener_s1 overlapped least as seen in Figure 6a1,a2. However, the pdf pairs related to Max_f_s1 had the maximum overlapping as in Figure 6c1–c3. A close look at Table 6 and Table 7 showed that the entropy measures were ranked in almost the same order. This evidence showed that the significance level drawn from the Mann–Whitney U test and the pdf overlapping were consistent and compatible. This proved that the Mann–Whitney U test was a useful tool to find effective entropy measures for identification purposes.

### 3.3. Identification Performance of Two Joint-Entropy Measures

**Figure 8:**
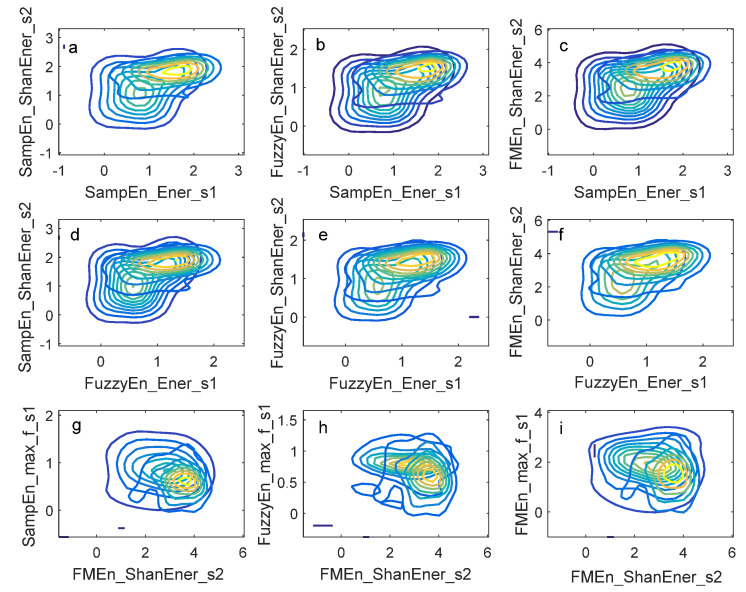
Contour plot of the joint pdf of two entropy measures. (**a**–**f**) The joint pdfs of the best six combinations; (**g**–**i**) the joint pdfs of the worst three combinations.

To improve identification performance, it is natural to use joint pdf of two selected entropy measures. A selection of any two measures out of the nine measures yields 36 combinations. The authors investigated the identification performance of the combinations one by one based on leave-one-out validation. The joint pdfs of the best six and the worst three combinations were shown in Figure 8. So, a subject can be classified as a PH patient or a healthy subject by Algorithm 2 based on the joint pdf proposed in Section 2.6 where the entropy measure vector is a two-dimensional vector. Visual observation to Figure 8a–f indicated the joint pdf of PH patients and healthy subjects had less overlapping where the peaks can be seen separately and clearly. However, the joint pdfs in Figure 8g–i overlapped to a high degree and the peaks were much closer than those in Figure 8a–f. So, a conclusion could be drawn from the observation that the identification performance of the joint measures of Figure 8a–f was much better than those in Figure 8g–i. The quantitative performance indicators of the nine combinations were shown in Table 8. The nine joint measures in Table 8 corresponded to the joint pdfs in Figure 8a–i. The best performance was achieved by the joint of SampEn_Ener_s1 and SampEn_ShanEner_s2 which resulted in that the sensitivity, specificity, accuracy and AUC were 0.680, 0.796, 0.740, and 0.770. The quantitative performance in Table 8 confirmed the observation in Figure 8. Comparison between Table 7 and Table 8 revealed that the identification performance got improvement by joint pdf of two entropy measures.

### 3.4. Identification Performance of the Joint pdf of Multiple Entropy Measures

Following the reasoning of the joint pdf of two entropy measures, the authors tried to use joint multiple measures to obtain better identification performance. Similarly, the joint pdf of multiple measures of PH group and health group can be built by the KDE estimation based on leave-one-out cross-validation. Then, the proposed Algorithm 2 was used to identify a subject as a patient or a healthy one where the entropy measure vector was a multidimensional vector. Unfortunately, the multidimensional pdfs cannot be visualized normally. The authors investigated the identification performance of all possible combinations of the entropy measures. The top ten results were shown in Table 9. These results were better than those in Table 8 (joint pdf of two entropy measures) and Table 7 (pdf of a single entropy measure). For example, in the first line in Table 9, the joint five entropy measures yielded the best identification performance with sensitivity of 0.740, specificity of 0.870, accuracy of 0.808, and AUC of 0.829. Similar excellent performance can also be obtained by other combinations, such as line 2 to line 10 in Table 9. A careful check to Table 8 and Table 9 revealed that the identification performance can be improved by joint multiple measures, as the authors expected.

### 3.5. Summary and Discussions

Pulmonary hypertension (PH) is often diagnosed late because early identification is very difficult even after the onset of symptoms. Therefore, early diagnosing the clinical PH is needed. Heart sound, acoustic vibration generated by the interaction between the heart hemodynamics and valves, chambers and great vessels, is an important physiological signal needed exploration for PH detection further. In the previous studies, four papers have studied the similar topic [8,9,10,11]. The four papers analyzed multiple heart sound features for normal subjects and patients. But, three studies [8,9,10] were for children and only one study [11] was for adults. The classification performance for children showed a sensitivity of 0.93 and a specificity of 0.92. The identification accuracy from the fourth paper for adults was 0.77. The authors verified for adults that the regularity of heart sound features of a PH patient varied comparing to that of a healthy subject. Sample entropy, fuzzy entropy and fuzzy measure entropy were calculated for the proposed 11 feature sequences. The identification was achieved by the difference of entropy measure probability occurred in PH group and health group. The results showed that the difference in entropy measure probability between the PH group and health group did exist. A subject can be classified by the estimated pdf based on the Bayes’ decision rule and the classification performance could be improved by joint entropy measures.

The authors wish to emphasize that the pdf was estimated based on leave-one-out cross-validation. In other words, only one subject was taken out and the remainders were involved in pdf training and then the taken-out subject was identified by the Bayes’ decision rule. On the other hand, the heart sound signals of PH patients or healthy subjects were all collected at the left third intercostal space. However, the heart sound signals collected from other sites were not considered in this study.

## 4. Conclusions

This study used the entropy measures (SampEn, FuzzyEn and FuzzyMEn) to evaluate the regularity of the proposed heart sound features of people with and without PH. The detection by Mann–Whitney U test found that the entropy measures related to, Max_pow_s1, Max_pow_s2, Max_f_s1, Max_f_s2, Ener_s1, Ener_s2, ShanEner_s1, ShanEner_s2, Car_cycle, Int_s2 were significant between the PH group and health group. The entropy measures related to Int_s1 were not significant. On the other hand, we conducted correlation analysis between an entropy measure and age value to reduce the age confounding factor. Nine entropy measures were selected as effective measures for identification purpose. Further, the pdf of each single entropy measure or the joint pdf of combined entropy measures were built by KDE estimation. Identification was achieved by the proposed Algorithm 1 and Algorithm 2 based on Bayes’ decision rule. In the case of single measure, the simulation result showed that SampEn_Ener_s1 yielded the best identification performance where the sensitivity and specificity were 0.720 and 0.648. Meanwhile, in the case of joint two measures, the identification performance was improved by joint pdf of SampEn_Ener_s1 and SampEn_ShanEner_s2 where the sensitivity and specificity were 0.680 and 0.796. Multiple measures were also combined to improve identification performance. The simulation results showed that a combination of five out of nine measures performed best where the sensitivity and specificity were up to 0.740 and 0.870. The identification process proposed in this manuscript could have potential application in early screening for PH.

## Figures and Tables

**Table 1:** Basic information of the PH patient group.

Item	Value	Range
Number (M/F)	50 (26/24)	-
Age (year)	69.4 ± 12.3	33–89
Height (cm)	164.0 ± 8.4	148–177
Weight (kg)	64.5 ± 12.6	32–90
BMI (kg/m^2^)	23.9 ± 4.3	14.2–35.3
PSBP (mmHg)	38.4 ± 11.8	20.4–68.0

Note: values are expressed as number (male/female) or mean ± standard deviation. BMI: body mass index; PSBP: pulmonary systolic blood pressure.

**Table 2:** Basic information of the healthy control group.

Item	Value	Range
Number (M/F)	54 (47/7)	-
Age (year)	32.6 ± 14.9	22–67
Height (cm)	172.5 ± 7.0	155–184
Weight (kg)	64.3 ± 7.8	43–76
BMI (kg/m2)	21.6 ± 2.3	17.6–26.9
PSBP (mmHg)	<25	-

Note: values are expressed as number (male/female) or mean ± standard deviation. BMI: body mass index; PSBP: pulmonary systolic blood pressure.

**Table 3:** Summary of the features.

No.	Name	Physical Meaning
1	Int_s1	Time interval of S1
2	Int_s2	Time interval of S2
3	Car_cycle	Cardiac cycle
4	Max_pow_s1	Maximum magnitude of the power spectral density of S1
5	Max_f_s1	The frequency value corresponding to “Max_pow_s1”
6	Max_pow_s2	Maximum magnitude of the power spectral density of S2
7	Max_f_s2	The frequency value corresponding to “Max_pow_s2”
8	Ener_s1	Average energy of S1
9	Ener_s2	Average energy of S2
10	ShanEner_s1	Average Shannon energy of S1
11	ShanEner_s2	Average Shannon energy of S2

**Table 4:** Identification algorithm using a single entropy measure.

**Algorithm 1:** identification using a single entropy measure
Let ${\hat{f}}_{p}(e)$ be the pdf of an entropy measure of the PH patient group and ${\hat{f}}_{h}(e)$ be the pdf of the entropy measure of the health control group. The entropy measure of an unknown subject is $e_{u}$. If ${\hat{f}}_{p}(e_{u})>{\hat{f}}_{h}(e_{u})$ then the unknown subject is judged as a PH patient else the unknown subject is judged as a healthy subject

**Table 5:** Identification algorithm using joint entropy measures.

**Algorithm 2**: identification using joint entropy measures
Let $\hat{f_{p}^{d}}(e)$ be the pdf of joint entropy measures of the PH patient group and $\hat{f_{h}^{d}}(e)$ be the joint pdf of the entropy measures of the health control group. The entropy measure vector of an unknown subject is $e_{u}$. If $\hat{f_{p}^{d}}(e_{u})>\hat{f_{h}^{d}}(e_{u})$ then the unknown subject is judged as a PH patient else the unknown subject is judged as a healthy subject

**Table 6:** List of the significance levels and correlation coefficients with age of the thirty three measures.

No.	Entropy Measure	*p* Value	CC.	No.	Entropy Measure	*p* Value	CC.
**1**	**SampEn_Ener_s1**	**1.49 × 10^−5^**	**−0.30**	18	SampEn_ShanEner_s1	9.79 × 10^−7^	−0.37
**2**	**FuzzyEn_Ener_s1**	**2.12 × 10^−5^**	**−0.29**	19	FuzzyEn_ShanEner_s1	3.67 × 10^−6^	−0.35
3	FMEn_Ener_s1	6.42 × 10^−6^	−0.33	20	FMEn_ShanEner_s1	1.60 × 10^−6^	−0.38
**4**	**SampEn_ShanEner_s2**	**5.88 × 10^−5^**	**−0.28**	21	FMEn_max_f_s2	1.52 × 10^−6^	0.41
**5**	**FuzzyEn_ShanEner_s2**	**6.57 × 10^−5^**	**−0.27**	22	SampEn_Ener_s2	1.11 × 10^−5^	−0.31
**6**	**FMEn_ShanEner_s2**	**1.29 × 10^−4^**	**−0.26**	23	FuzzyEn_Ener_s2	1.76 × 10^−5^	−0.31
**7**	**SampEn_max_f_s1**	**7.25 × 10^−4^**	**0.21**	24	FMEn_Ener_s2	3.36 × 10^−5^	−0.31
**8**	**FuzzyEn_max_f_s1**	**3.52 × 10^−3^**	**0.14**	25	SampEn_max_pow_s1	1.58 × 10^−5^	−0.40
**9**	**FMEn_max_f_s1**	**6.47 × 10^−3^**	**0.16**	26	FuzzyEn__max_pow_s1	1.14 × 10^−5^	−0.40
10	SampEn_Car_cycle	4.90 × 10^−3^	−0.31	27	FMEn_max_pow_s1	3.91 × 10^−6^	−0.40
**11**	**FuzzyEn_Car_cycle**	**3.99 × 10^−3^**	**−0.30**	28	SampEn_Int_s2	5.38 × 10^−4^	0.39
12	FMEn_Car_cycle	1.89 × 10^−3^	−0.33	29	FuzzyEn_Int_s2	5.06 × 10^−4^	0.42
13	SampEn_max_pow_s2	1.20 × 10^−9^	−0.41	30	FMEn_Int_s2	9.26 × 10^−3^	0.37
14	FuzzyEn__max_pow_s2	2.69 × 10^−9^	−0.39	31	SampEn_Int_s1	4.22 × 10^−1^	0.21
15	FMEn_max_pow_s2	6.13 × 10^−9^	−0.39	32	FuzzyEn_Int_s1	2.35 × 10^−1^	0.22
16	SampEn_max_f_s2	8.02 × 10^−7^	0.43	33	FMEn_Int_s1	3.92 × 10^−1^	0.19
17	FuzzyEn_max_f_s2	4.27 × 10^−5^	0.39				

Note: the bold format represents the selected entropy measures.

**Table 7:** Identification performance of a single entropy measure based on leave-one-out cross-validation.

No.	Entropy Measure	Sen.	Spe.	Acc.	AUC	Corresponding pdf Pair and ROC Curve
1	SampEn_Ener_s1	0.720	0.648	0.683	0.720	Figure 6a1 and Figure 7a1
2	FuzzyEn_Ener_s1	0.680	0.648	0.663	0.714	Figure 6a2 and Figure 7a2
3	FuzzyEn_Car_cycle	0.600	0.852	0.731	0.709	Figure 6a3 and Figure 7a3
4	SampEn_ShanEner_s2	0.580	0.796	0.692	0.667	Figure 6b1 and Figure 7b1
5	FuzzyEn_ShanEner_s2	0.480	0.778	0.635	0.681	Figure 6b2 and Figure 7b2
6	FMEn_ShanEner_s2	0.500	0.796	0.654	0.670	Figure 6b3 and Figure 7b3
7	SampEn_Max_f_s1	0.540	0.759	0.654	0.629	Figure 6c1 and Figure 7c1
8	FuzzyEn_Max_f_s1	0.660	0.648	0.654	0.646	Figure 6c2 and Figure 7c2
9	FMEn_Max_f_s1	0.580	0.667	0.625	0.584	Figure 6c3 and Figure 7c3

Sen.: sensitivity; Spe: specificity; Acc.: accuracy.

**Table 8:** Identification performance of joint pdf of two entropy measures based on leave-one-out cross-validation.

No.	Joint Two Entropy Measures	Sen.	Spe.	Acc.	AUC	Corresponding pdfs
1	SampEn_Ener_s1/SampEn_ShanEner_s2	0.680	0.796	0.740	0.770	Figure 8a
2	SampEn_Ener_s1/FuzzyEn_ShanEner_s2	0.680	0.796	0.740	0.759	Figure 8b
3	SampEn_Ener_s1/FMEn_ShanEner_s2	0.640	0.778	0.712	0.756	Figure 8c
4	FuzzyEn_Ener_s1/SampEn_ShanEner_s2	0.640	0.778	0.712	0.759	Figure 8d
5	FuzzyEn_Ener_s1/FuzzyEn_ShanEner_s2	0.640	0.759	0.702	0.748	Figure 8e
6	FuzzyEn_Ener_s1/FMEn_ShanEner_s2	0.620	0.778	0.702	0.741	Figure 8f
7	FMEn_ShanEner_s2/SampEn_Max_f_s1	0.540	0.704	0.625	0.680	Figure 8g
8	FMEn_ShanEner_s2/FuzzyEn_Max_f_s1	0.580	0.648	0.615	0.680	Figure 8h
9	FMEn_ShanEner_s2/FMEn_Max_f_s1	0.520	0.722	0.625	0.666	Figure 8i

**Table 9:** Top ten identification performance of joint pdf of multiple entropy measures based on leave-one-out cross-validation.

No.	Joint Entropy Measures	Number of Joint Measures	Sen.	Spe.	Acc.	AUC
1	SampEn_Ener_s1/FuzzyEn_Ener_s1/SampEn_Max_f_s1/FuzzyEn_Car_cycle/FMEn_Max_f_s1	5	0.740	0.870	0.808	0.829
2	SampEn_Ener_s1/SampEn_Max_f_s1/FuzzyEn_Max_f_s1/FuzzyEn_Car_cycle	4	0.740	0.870	0.808	0.814
3	SampEn_Ener_s1/SampEn_Max_f_s1/FuzzyEn_Car_cycle/FMEn_Max_f_s1	4	0.740	0.870	0.808	0.813
4	SampEn_Ener_s1/FuzzyEn_Ener_s1/SampEn_Max_f_s1/FuzzyEn_Car_cycle	4	0.720	0.870	0.798	0.839
5	SampEn_Ener_s1/FuzzyEn_Ener_s1/SampEn_Max_f_s1/FuzzyEn_Max_f_s1/FuzzyEn_Car_cycle/FMEn_Max_f_s1	6	0.740	0.852	0.798	0.810
6	SampEn_Ener_s1/FuzzyEn_Car_cycle/FMEn_Max_f_s1	3	0.720	0.870	0.798	0.798
7	SampEn_Ener_s1/SampEn_Max_f_s1/FuzzyEn_Car_cycle	3	0.720	0.852	0.788	0.821
8	SampEn_Ener_s1/FuzzyEn_Ener_s1/SampEn_Max_f_s1/FuzzyEn_Max_f_s1/FuzzyEn_Car_cycle	5	0.760	0.815	0.788	0.818
9	SampEn_Ener_s1/SampEn_Max_f_s1/FuzzyEn_Max_f_s1/FuzzyEn_Car_cycle/FMEn_Max_f_s1	5	0.720	0.833	0.779	0.801
10	FuzzyEn_Ener_s1/SampEn_Max_f_s1/FuzzyEn_Car_cycle	3	0.680	0.852	0.769	0.815

## Data Availability

The data used in this study is available to those who wish to reproduce the results.
